# Environmental Impact on Seaweed Phenolic Production and Activity: An Important Step for Compound Exploitation

**DOI:** 10.3390/md19050245

**Published:** 2021-04-26

**Authors:** Silvia Lomartire, João Cotas, Diana Pacheco, João Carlos Marques, Leonel Pereira, Ana M. M. Gonçalves

**Affiliations:** 1 University of Coimbra, MARE-Marine and Environmental Sciences Centre, Department of Life Sciences, Calçada Martim de Freitas, 3000-456 Coimbra, Portugal; silvia.lomartire@uc.pt (S.L.); jcotas@uc.pt (J.C.); diana.pacheco@uc.pt (D.P.); jcmimar@ci.uc.pt (J.C.M.); leonel.pereira@uc.pt (L.P.); 2 Department of Biology and CESAM, University of Aveiro, 3810-193 Aveiro, Portugal

**Keywords:** seaweeds, phenolic compounds, bioactive compounds, pharmaceutical application, nutraceutical application

## Abstract

Seaweeds are a potential source of bioactive compounds that are useful for biotechnological applications and can be employed in different industrial areas in order to replace synthetic compounds with components of natural origin. Diverse studies demonstrate that there is a solid ground for the exploitation of seaweed bioactive compounds in order to prevent illness and to ensure a better and healthier lifestyle. Among the bioactive algal molecules, phenolic compounds are produced as secondary metabolites with beneficial effects on plants, and also on human beings and animals, due to their inherent bioactive properties, which exert antioxidant, antiviral, and antimicrobial activities. The use of phenolic compounds in pharmaceutical, nutraceutical, cosmetics, and food industries may provide outcomes that could enhance human health. Through the production of healthy foods and natural drugs, bioactive compounds from seaweeds can help with the treatment of human diseases. This review aims to highlight the importance of phenolic compounds from seaweeds, the scope of their production in nature and the impact that these compounds can have on human and animal health through nutraceutical and pharmaceutical products.

## 1. Introduction

Seaweeds have been found to be very versatile natural sources of several metabolites [1]. Different studies demonstrate that algal bioactive compounds can be used in many applications, such as the production of healthy food [2] and pharmaceutical [3] and cosmetic products [4]. Seaweed compounds are exploited for the production of food, feed, cosmetic, and fertilizers [5,6,7,8,9]. According to the data presented in the literature, seaweeds contain a low fat and lipids (polyunsaturated fatty acids) content and a high concentration of polyphenols, carbohydrates, proteins, minerals, vitamins, and pigments [10,11]. Despite the chemical composition of seaweeds not being as known as that of terrestrial plants, it has been demonstrated that some biological compounds are only produced by marine algae [12]. For this reason, they are investigated for their use in biotechnological applications. Among the secondary metabolites produced by plants and seaweeds, phenolic compounds possess several properties that make them interesting for medical, nutraceutical and industrial purposes [13,14,15]. Phenolic compounds have the potential to positively influence the human lifestyle in the development of drugs with therapeutical activities [16,17,18]. Most plants, including seaweeds, produce polyphenols [19]. The most common polyphenols are phenolic acids, tannic acids, flavonoids, isoflavones, cinnamic acid, benzoic acid, quercetin, and lignans [17,20].

Phenolic compounds also contribute for the color of the seaweeds [21], furthermore they developed several biological activities. These compounds are synthesized as a defense mechanism: they protect plants and seaweeds from stress conditions [22,23], their antibacterial activity reduces diseases [23] and they play a role in herbivore defense [24,25]. Polyphenols prevent seaweeds from the colonization of microalgae, bacteria, fungi, and small invertebrates that may affect the physiology of seaweeds [26,27]. Among phenolic compounds, phlorotannins are common in brown seaweeds and these compounds play pivotal structural and protective roles [28]. For example, Le Lann et al. [29] noticed a more intense production of phenolic compounds during the reproductive period in Sargassaceae (Phaeophyceae, brown algae), likely as a response to ultraviolet (UV) radiation [30]. The powerful activities exerted by phenolic compounds make them useful for different applications; their assimilation by humans provides several health benefits [31]. Results demonstrate that foods and beverages enriched with polyphenolic compounds may reduce cardiovascular diseases [32,33]. In addition, phlorotannins show antimicrobial, cytotoxic, antioxidant, and antitumoral activities, and due to their complex structures, they are also strong free radical scavengers [28,34,35,36].

Due to the increasing interest regarding algal phenolic compounds, this review focuses on their biological activities, highlighting the importance of phenolic compounds in seaweeds, but also how they may be exploited in biotechnological and industrial applications.

## 2. Seaweed Phenolic Compounds

Seaweed phenolic compounds are attracting the attention of the scientific community, as well as several industries, due to their high variety and potential uses [14,37,38]. For instance, the occurrence of phlorotannins (in brown seaweeds) and bromophenols, flavonoids, phenolic terpenoids, and mycosporine-like amino acids (MAAs) in green and red seaweeds has been recorded (Table 1) [39,40].

Phenolic acids consist of a single phenol ring and at least a group of functional carboxylic acids and are typically graded according to the number or the amount of carbon in the chain bound to the phenolic ring. These phenolic acids are also categorized as C6-C1 for hydroxybenzoic acid (HBA; one carbon chain linked to the phenolic ring), C6-C2 for acetophenones and phenylacetic acids (two carbon chains linked to the phenolic ring) and C6-C3 (3 carbon chains attached to the phenol ring) for hydroxycinnamic acid (HCA) [41,42]. HBAs include, among others, gallic acid, p-hydroxybenzoic acid, vanillic acid, syringic acid, and protocatechins, in which there are differences in the basic structure of the HBA, including an aromatic ring hydroxylation and methoxylation [41,42].

Trans-phenyl-3-propenoic acids are hydroxycinnamic acids (HCA), which vary in their ring constitution [41]. These HCA derivatives include caffeic (3,4-dihydroxycinnamic), ferulic (3-methoxy-4-hydroxy), sinapic (3,5-dimethoxy-4-hydroxy), and p-coumaric (4-hydroxy) acids, all of which are commonly distributed as conjugates, primarily as quinic acid esters (chlorogenic acids) [41,42]. In addition, these acids can be subcategorized up into different groups based on the identity, location, and number of the acyl residue: (1) mono-esters of caffeic, ferulic, and p-coumaric acids; (2) bi-, tri-, and tetra-esters of caffeic acids; (3) mixed di-esters of caffeic-ferulic acid or caffeic-sinapic acids; and (4) mixed caffeic acid esters with aliphatic dibasic acids, such as oxalic or succinic acid [41,42].

**Figure 1:**
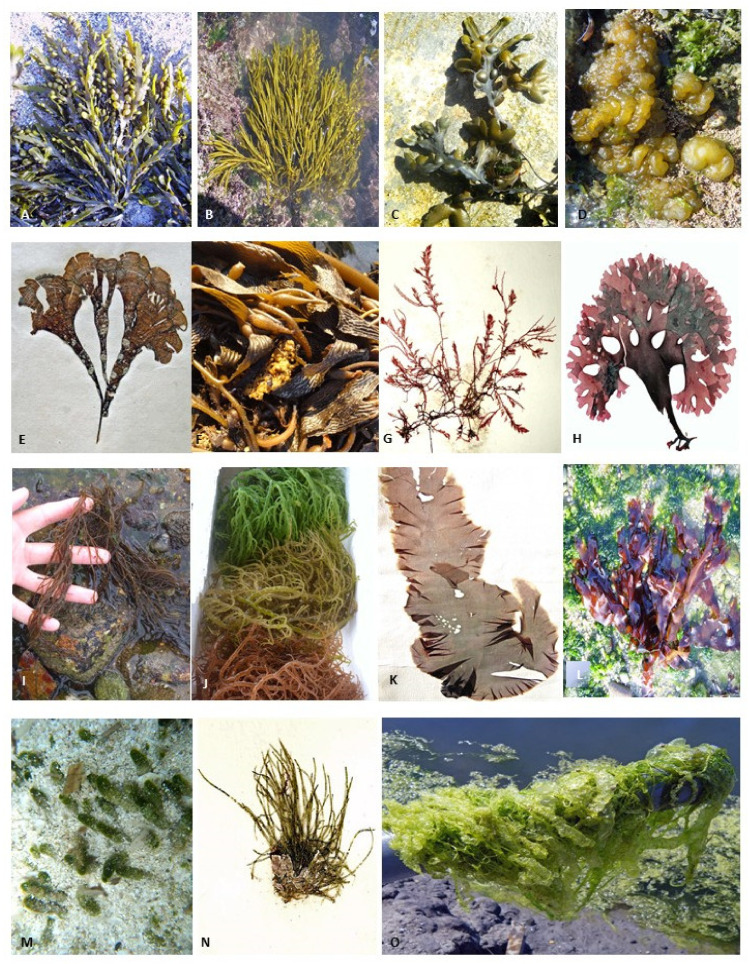
Some seaweeds producing phenolic compounds: (**A**)—*Ascophyllum nodosum* (P); (**B**)—*Bifurcaria bifurcata* (P); (**C**)—*Fucus vesiculosus* (P); (**D**)—*Leathesia marina* (P); (**E**)—*Lobophora variegata* (P); (**F**)—*Macrocystis pyrifera* (P); (**G**)—*Asparagopsis armata* (R); (**H**)—*Chondrus crispus* (R); (**I**)—*Gracilaria* sp. (R); (**J**)—*Kappaphycus alvarezii* (R); (**K**)—*Neopyropia* sp. (R); (**L**)—*Palmaria palmata* (R); (**M**)—*Dasycladus vermicularis* (Chl); (**N**)—*Derbesia tenuissima* (Chl); (**O**)—*Ulva intestinalis* (Chl); *P—Phaeophyceae*, R—Rhodophyta; Chl—Chlorophyta.

Some experiments have shown the presence of phenolic acids in marine algae [41,42,43]. For instance, coumarins have been found in green seaweed species such as *Dasycladus vermicularis*, as well as some vanillic acid derivatives in the *Cladophora socialis* (Chlorophyta, green algae) [44]. *Ascophyllum nodosum* (Figure 1A), *Bifurcaria bifurcata* (Figure 1B), and *Fucus vesiculosus* (Figure 1C) (Phaeophyceae, brown algae) have been distinguished by the presence of HBAs, rosmarinic acid, and quinic acid [45]. In addition, in the genus *Gracilaria* (Figure 1I) (Rhodophyta, red alga), phenolic acids have been detected, such as benzoic acid, p-hydroxybenzoic acid, salicylic acid, gentisic acid, protocatechuic acid, vanillic acid, gallic acid, and syringic acid [46,47,48].

Phlorotannins are well-known phenolic compounds synthesized by brown seaweeds. These compounds are constituted by oligomeric units of phloroglucinol [49,50]. Commonly, these secondary metabolites have a molecular weight ranging from 10 to 100 kDa, due to the high variability that these molecules can present in the structural bonds between phloroglucinol and the hydroxyl groups [51,52]. In this context, phlorotannins can be categorized into six categories: (1) fucols (aryl–aryl bonds), (2) phloretols (aryl–ether bonds), (3) eckols (dibenzo-1,4-dioxin bonds), (4) fucophloretols (ether or phenyl linage), (5) carmalols (dibenzodioxin moiety), and (6) fuhalols (ortho-/para- arranged ether bridges containing an additional hydroxyl group on one unit) [49,51,52]. Moreover, the complexity of these molecules classify them, by each category, into linear or branched phlorotannins [51,52]. Due to its biotechnological properties, dieckol is the most exploited phlorotannin, and it can be found in the species *Ecklonia cava* (Phaeophyceae) [53].

Flavonoids are structurally characterized as phenolic compounds with a heterocyclic oxygen bound to two aromatic rings, which can then differ according to the degree of hydrogenation [54,55]. However, there is a generalized lack of studies regarding algal flavonoids’ isolation and characterization. Nevertheless, some research has shown that seaweeds are a rich source of flavonoids. Several species of the Chlorophyta, Rhodophyta phyla, and Phaeophyceae class were found to have flavonoids such as rutin, quercitin, and hesperidin [49,56]. For instance, *Chondrus crispus* (Figure 1H) and *Porphyra*/*Pyropia* spp. (Rhodophyta) and *Sargassum muticum* and *Sargassum vulgare* (Phaeophyceae) can synthesize isoflavones, likewise daidzein or genistein [57]. Moreover, many flavonoid glycosides have also been recorded in the brown seaweeds *Durvillaea antarctica*, *Lessonia spicata*, and *Macrocystis pyrifera* (also known as *Macrocystis integrifolia*) (Figure 1F) [49]. Furthermore, green (*Acetabularia ryukyuensis*), brown (*Eisenia bicyclis*—as *Ecklonia bicyclis*, *Padina arborescens*, *Padina minor*), and red seaweeds (*Neopyropia yezoensis*—also known as *Porphyra yezoensis*—Figure 1K, *Gelidium elegans*, and *Portieria hornemannii*—also known as *Chondrococcus hornemannii*) proved to be a valuable source of catechin, epicatechin, epigallocatechin, catechin gallate, epicatechin gallate, or epigallocatechin gallate [58].

Bromophenols are brominated phenolic compounds characterized by the presence of one or more benzene rings and hydroxyl substituents [59,60]. These compounds can be found in green [61,62,63,64], red [65,66,67] and brown seaweeds [68,69]. Nevertheless, red seaweeds often exhibit a higher content of these molecules [70]. However, due to the low content of bromophenols in seaweeds, there are just a few studies regarding the isolation and characterization of these compounds.

Phenolic terpenoids are secondary metabolites that have already been identified in seaweeds [50]. For instance, meroditerpenoids (such as plastoquinones, chromanols, and chromenes) were found in brown seaweeds, mainly from the family Sargassaceae (Phaeophyceae). These compounds are partially derived from terpenoids and are characterized for having a polyprenyl chain linked to a hydroquinone ring moiety [71]. Red seaweeds also synthesize phenolic terpenoids, such as diterpenes and sesquiterpenes in Rhodomelaceae. For example, the species *Callophycus serratus* synthesizes a specific diterpene, bromophycolide [72].

**Table 1:** Seaweed phenolic compounds recorded, according to phyla and phenolic compound group.

Seaweed Species	Phenolic Compound Group	Compound	Reference
**Chlorophyta**			
*Acetabularia ryukyuensis* *Dasycladus vermicularis*	Flavonoids	Catechin, epicatechin, epigallocatechin, catechin gallate, epicatechin gallate, or epigallocatechin gallate Coumarin	[44,61]
*Dasycladus vermicularis* *Cladophora socialis* *Nitella hookeri*	Phenolic acids Flavonoids	Coumarin Vanillic acid C-glycosides	[44,58]
**Rhodophyta**			
*Gracilaria* sp.	Phenolic acids	Benzoic acid, p-hydroxybenzoic acid, salicylic acid, gentisic acid, protocatechuic acid, vanillic acid, gallic acid, and syringic acid	[47]
*Chondrus crispus*	Flavonoids	Isoflavones; daidzein or genistein	[57]
*Porphyra*/*Pyropia* spp.		Isoflavones; daidzein or genistein	
*Neopyropia yezoensis* (as *Porphyra yezoensis*)		Catechin, epicatechin, epigallocatechin, catechin gallate, epicatechin gallate, or epigallocatechin gallate	[58]
*Gelidium elegans*			
*Portieria hornemannii* (as *Chondrococcus hornemannii*)			
*Callophycus serratus*	Phenolic terpenoids	Bromophycolides	[72]
*Palmaria palmata*	Mycosporine-like amino acids	Palythine, shinorine, asterina-330, palythinol, and porphyra-334	[73]
*Falkenbergia rufolanosa* (tetrasporophyte phase of *Asparagopsis armata*)		Palythine and shinorine	[74]
**Ochrophyta, Phaeophyceae**			
*Ascophyllum nodosum*	Phenolic acids	Rosmarinic acid; quinic acid	[45]
*Bifurcaria bifurcata*			
*Fucus vesiculosus*			
*Ecklonia cava* *Cystoseira* sp. *Fucus spiralis* *Ishige okamurae* ***Ascophyllum nodosum*** ***Bifurcaria bifurcata***	Phlorotannins	Dieckol Eckol Fucophloroethol-type Diphlorethohydroxycarmalol Fucaphlorethol-type Tetrafuhalol B	[53,75,76,77,78]
*Durvillaea antarctica*	Flavonoids	C-glycosides	[49]
*Lessonia spicata*			
*Macrocystis pyrifera* (as *Macrocystis integrifolia*)			
*Eisenia bicyclis* (as *Ecklonia bicyclis*) *Padina arborescens* *Padina minor*		Catechin, epicatechin, epigallocatechin, catechin gallate, epicatechin gallate, or epigallocatechin gallate	[58]
*Sargassum muticum*		Daidzein or genistein	[57]
*Sargassum vulgare*			
Sargassaceae	Phenolic terpenoids	Plastoquinones, chromanols, and chromenes	[71]
*Stypopodium zonale*		Stypofuranlactone; 10,18-dihydroxy-5′a-desmethyl-5′-acetylatomaric acid; 10-keto-10-deisopropyliden-5′a-desmethyl-5′-acetylatomaric acid; 10-keto-10-deisopropyliden-atomaric acid	[79]

Mycosporine-like amino acids (MAAs) are secondary metabolites that, despite being synthesized by several organisms, were found to be more often produced by marine organisms [80,81,82]. Such compounds present a low molecular weight (<400 kDa) and are soluble in water. Moreover, they present a cyclohexanone or cyclohexenine ring, with amino acid moieties in their chemical structure [80,83]. Thus, these compounds can be found mainly in red seaweeds. For example, it was found that the edible red seaweed *Palmaria palmata* (Figure 1L) biosynthesizes the MAA palythine, shinorine, asterina-330, palythinol, and porphyra-334 [73]. In addition, the tetrasporophyte phase of *Asparagopsis armata* (Figure 1G) was found to produce palythine and shinorine [74].

## 3. Drivers to the Production of Phenolic Compounds and Their Impact on Bioactivity

Phenolic compound production in nature is typically triggered by extrinsic or intrinsic drivers. The phenolic compounds (primary and secondary metabolites) have a natural and inherent initial production (primary metabolites), in basic conformations. The more complex forms are produced when seaweed cells are triggered under stress conditions [15]. Consequently, phenolic compounds presence is always detected in cells [15]. However, extrinsic factors trigger cellular defensive responses, which can shift the molecular mechanism to produce higher quantities and a wider range of conformations of a specific compound class, mainly when it is a defensive compound, which is synthesized to protect algae from external attacks [84,85]. Phenolic compounds detected in seaweeds present differences between the red, green, and brown seaweeds phenolic compounds. Different phyla produce different compounds, e.g., phlorotannins are only produced by brown seaweeds and red seaweeds produce a wide variety of MAAs in higher levels than green species [15]. Thus, the phenolic compounds production and its diversity are directly related to the seaweed taxonomic group and to specific species, due to cellular mechanism and genetic codification [50].

In this section, quality refers to the high diversity of compounds, thus seaweeds bioactivities can be different according to the phenolic compounds’ concentration and respective characterization. Therefore, the overall quantity of phenolic compounds in seaweeds can change, without changing the phenolic compounds diversity, yet its bioactivity can be different [86,87]. On the other hand, the profile of seaweed phenolic compounds can change, impacting the respective bioactivity, which depends on intrinsic and extrinsic factors affecting seaweeds [88].

### 3.1. Drivers to the Phenolic Compounds Production

There are intrinsic drivers in seaweeds’ DNA and codifications, which may limit the natural production of phenolic compounds. There are large differences in phenolic compounds produced by red, green, and brown seaweeds, even in their natural quantity between species [15,70] and their inherent bioactivities. Furthermore, other intrinsic factors are species, life stage, size, age, thallus morphology, and reproductive status [89,90].

There are requirements at a cellular level to produce certain phenolic compounds. For example, phlorotannins need an active production of phloroglucinol to synthesize phloroglucinol oligomers derivatives [15,49]. Pathways for bromophenol synthesis are still unclear; however, for their biosynthesis pathways, the presence of bromoperoxidases, bromase, laccase, hydrogen peroxide, and bromide, at least, is needed [15,63,70,91,92]. Furthermore, this can be correlated with the concentration of photosynthetic pigments in seaweeds [92,93]. Regarding flavonoids, there are a few contradictory reports in the literature, in which the intrinsic factors for flavonoids production are not explained, and the same is true for phenolic terpenoids and phenolic derivates, MAAs, in the red seaweeds [15]. However, there are similarities between all the phenolic compounds produced by the seaweeds, which is the primary metabolic pathways, which are commonly the shikimate and/or acetate pathway [15,94,95]. Despite recent developments, the impacts of intrinsic and extrinsic factors remain unclear, and represent a roadblock to further investigation and exploitation of the interesting aspects of phenolic compounds at an industrial scale [15,70].

### 3.2. Extrinsic Factors and Their Impact in the Phenolic Compounds Production and Activity

Phenolic compounds can be produced in high quantity and/or quality, due to the direct impact of environmental extrinsic factors [84,85]. Extrinsic impacts in the seaweed phenolic compounds quality and quantity include seaweed geolocation, ecological characterization, season, biotic factors (herbivory or direct competition with other benthic organisms) and abiotic factors (salinity, pH, light incidence, temperature, and water nutrient composition) [15,84,96]. Depending on the species, season, and location, the literature describes different impacts in the phenolic compounds’ total concentration and profile [97,98,99,100]. This happens because seaweed habitats are characterized by complex and dynamic factors, with the occurrence of extreme changes in the surrounding environment. These changes affect their chemical compounds concentration and composition greatly [84,101,102,103,104,105].

These factors can impact the diversity of the phenolic compounds produced or only their quantity. Cotas et al. [84] report that salinity did not have an effect in the improvement of the production of other phenolic compounds; however, it shifted the cellular mechanism to produce more pigments and phenolic compounds and reduce the production of polysaccharides. This work demonstrates that in *Fucus ceranoides*, the pigment quantity was the major difference in the overall bioactive assay. Thus salinity does not appear to affect the bioactivity (only the pigment: phenolic compounds ratio) [84,106]. However, the work of van Hees et al. [104] demonstrates that seaweed phenolic compounds in another location react positively to the salinity, in *Sirophysalis trinodis* and *Sargassopsis decurrens*. In light of this, there is a need to do a pro component analysis and a multiple factor analysis to have a foremost overview of the impact of the extrinsic factors in the seaweed phenolic compounds [104].

This needs to be done because of the different levels of impact of the intrinsic/extrinsic factors and reaction of the seaweed cellular mechanism to them, as demonstrated by Guihéneuf et al. [107] in the MAAs concentration in three red seaweeds *Palmaria palmata*, *Chondrus crispus*, and *Porphyra dioica*. This occurs mostly due to the presence of limiting factors (nutrients and light, mainly).

Another important factor in the production of phenolic compounds is the level of nutrients in the water: a higher nutrient content will reduce the quantity of phenolic compounds produced due to the seaweed focusing on growth, as demonstrated by Jormalainen et al. [108]. This study demonstrated that the high concentration of nutrients in seawater (nitrogen and phosphorus-based compounds) can diminish the phlorotannins yield in *Fucus vesiculosus*, although the same study identified that the diversity of phlorotannins is due mainly to the seaweed genotypic variation.

Contaminants and metals in the surrounding waters also impact the production of phenolic compounds in the brown seaweeds, where the lower molecular weight compounds (phlorotannins) are exuded in order to chelate these noxious compounds [30,109]. The study of Connan et al. [109] demonstrated that copper, zinc, cadmium, and chromium can be chelated by intra-cellular phenolic compounds and/or by polyphenols in the seaweed cell wall, in *Ascophyllum nodosum* and *Fucus vesiculosus*. This supports the phenolic activity as a natural detoxification mechanism to protect seaweed from contaminants, such as metals [33]. Moreover, this study also demonstrates the differences in the phlorotannin production and exudation with the low salinity gradient and evident differences in higher salinity. Consequently, there is a need to standardize the analytical techniques for metal concentration assessment in the algal biomass for human consumption, in order to guarantee a safe exploitation of these marine resources [1,5,6,7].

#### 3.2.1. Environmental Factors That Influence the Production of Phenolic Compounds

The phenolic compounds are produced in higher quantity if there is also a trigger modification, in which the seaweed needs to guarantee their survival, and this is also a seaweed reaction to external factors that represent life-threatening conditions/situations where the phenolic compounds have a direct interaction [15,70,92].

The phlorotannins are multifunctional compounds for seaweeds, especially as chemical defense against herbivory, anti-oxidant, and antifouling agents, thus when the seaweed is attacked by herbivores or when there is high light intensity, the phlorotannins are produced intensely to protect the seaweed from the external damage [110,111,112]. In the specific case of bromophenols, the extrinsic factor of tide emersion has a great impact on bromophenol quality, where the simple bromophenols units have high quantity in the species collected in the low tide, thus the effect of emergence of the seaweed from the seawater and exposure to the air; this happen in all the 49 species collected by Whitfield et al. [113]. In this case, the extrinsic factors have a high impact in the quality and quantity of bromophenols produced by seaweeds, which evidences great differences in the bioactivity [114].

The production of bromophenolic compounds is increased when the seaweeds (such as *Ulva lactuca*) are attacked or they sense a possible herbivore or nearby space occupation by other species, thus it is a chemical agent for defensive and deterrent action [15,61,70,92].

In the case of seaweed flavonoids and phenolic terpenoids, there is a general lack of literature; however, this can happen similarly to the bromophenol production, where only with a specific condition they are produced enough to be identified, due to also being secondary metabolites to protect seaweeds from herbivory [15,56,71].

The MAAs are enhanced in quantity and quality by the UV and light incidence. MAAs act mainly as UV-absorbing agents; consequently, these compounds actively protect the cells from UV-induced damage, such as the production of reactive oxygen species [15,50,83,115]. As demonstrated above by the work of Guihéneuf et al. [107], there are other reports that also link MAAs production to seawater nutrient concentrations, mostly nitrogen concentration, which appears to impact directly the MAAs’ concentration. [116,117,118]

Therefore, these factors have a high direct impact in the quantity and quality of the phenolic compounds, and thus a direct impact in the bioactivity that they further present.

#### 3.2.2. Extrinsic Factors to Promote Phenolic Compound Production: An Advantage to Exploit Further

A deeper knowledge about the main factors that alter quality and quantity of seaweed phenolic compounds is necessary to exploit their activities successfully. In the literature, the most studied and understood factor is the UV light incidence, which enhances a direct response of seaweed in the production of phenolic compounds to prevent oxidative stress and consequent cellular damage, thus under high light incidence (light quantity and quality), there is a high quantity and diversity of phenolic compounds [115,119,120]. Moreover, seaweed cellular mechanisms involve phenolic compounds to act in a dynamic photoinhibition [119,120]. In this case, with high UV light incidence, the phenolic compounds will have higher anti-oxidant capacity, when compared to the seaweeds under lower UV light incidence [115]. Thus, this abiotic factor is interesting to explore, enhancing the production of phenolic compounds to be further employed in human health-related products (foods, cosmetics, pharmaceutical, and medical) [121,122].

To cope with the harsh environmental conditions, algae synthesize and accumulate phenolic compounds [34]. For instance, grazing pressure is usually the lowest along wave-exposed coasts, where consumers’ feeding capacity is limited to calm times; as a result, greater water movement in the infralittoral zone makes grazing on algae more difficult than in the continuously submerged areas [123]. Previous research discovered that species belonging to Sargassaceae family synthesize phenolic compounds during the reproductive cycle, likely as a chemical defense against grazers, epiphytes, or UV radiation [29]. Thus, the grazing and epiphytes presence enhance the production of phenolic compounds in terms of quality and quantity [30,124,125]. For example, phlorotannin production does not stop as the thallus ages, but it does decrease as the thallus thickens and photosynthesis decreases [110].

However, this thematic has a general lack of literature and it is not exploited due to the high variables, which are bigger when compared to the natural abiotic factors and lower knowledge on how to put the seaweed in stress with herbivores pheromones or chemical trails without putting herbivores in the designed system to test their impact on seaweed phenolic compounds level and/or composition [30,110,124,125].

Moreover, there are several intrinsic and extrinsic factors with a high impact in the phenolic compound quantity and quality, e.g., the natural seasonal variation has a high impact in the wild seaweed specimen phenolic compounds. Due to this ecological impact, there is an uncertainty in the phenolic compounds’ composition and yield from wild seaweed, which makes them a risky raw ingredient for industrial exploitation, even with standard and safe extraction methods [15]. Despite this negative impact, there is ancient technology that has been evolving to the production of seaweed compounds, already with phenolic compounds explored commercially [1,15,126]. This technology is seaweed aquaculture, which has been evolving to guarantee seaweed quality and safety. Thus, the sustainable exploitation of seaweed phenolic compounds tends to be focused in the seaweed cultivation, due to their safety and production control (where cultivation will add a production cost and a positive safety). However, for the full exploitation of this technique, there is a need to develop cultivation methods from a multidisciplinary perspective. Preliminary cultivation studies can allow the control and understanding of the impact of extrinsic and intrinsic factors, in order to obtain a specific yield and quality of a certain seaweed compound, with a high rate of success, without putting pressure in the wild seaweed community while also reducing production costs [1,97,115,127,128,129,130,131,132,133,134,135,136,137,138,139,140,141,142,143,144,145].

### 3.3. Future Exploitation of the Phenolic Compounds

If the drivers of seaweed compounds production are fully understood, they can give more exploitation safety and information about how to efficiently explore the phenolic compounds, lowering the production costs and enhancing the compounds’ quality [1,115].

There is a general lack of preliminary studies on how abiotic and biotic factors affect the seaweed metabolism. The major gap in our knowledge relates to the relative importance of biotic and abiotic factors for phenolic compounds’ quality and quantity. Having the right acknowledgments will allow the exploitation of the seaweed phenolic compounds to be enhanced by the industry. That already happens; however, it is very limited when compared with the phenolic compounds’ potential [15]. Hence, the variability in quality and quantity reduces the interest of the phenolic compounds by the industry as a natural substitute to other raw ingredients, mostly synthetic [15].

Vega et al. [146] studied a pre-conception of aquaculture without different parameters from the normal use, using a traditional laboratory cultivation system, and the phenolic compounds diminished during the cultivation when compared to wild specimens. However, this impact can be due to the limiting factor related to the high presence of nitrogen in the media, as seaweed grows better with higher quantities of nitrogen compounds in the cultivation system, thus seaweeds do not produce as much phenolic compounds.

The study of Pedra et al. [147] demonstrates that *Kappaphycus alvarezii* cultivation with shrimp farming effluent can enhance the production of phenolic compounds and flavonoids. However, the phenolic compounds’ bioactivity is reduced when compared with commercial extract of the brown seaweed *A. nodosum*, which promotes lower phenolic compounds production which have higher bioactivity.

Pliego-Cortés [115] demonstrated a potential cultivation system to produce seaweed phenolic compounds when he analyzed three extrinsic factors (stress tolerance, nutrient concentration, and solar radiation) with the seaweed quantity and quality of a specific group of seaweed phenolic compounds (MAAs), and the overall seaweed phenolic compounds. They provided data and a PCA (Pro Component Analysis) detailed analysis of the impact of the abiotic factors on the content variation in MAAs. Moreover, this study also demonstrated the impact of the drivers’ relationship with the phenolic compounds bioactivity, leading to the possible exploitation of seaweed cultivation (under determined conditions) to obtain highly interesting molecules to be applied in food, cosmetic, and pharmaceutical products, with anti-oxidant and anti-tumor activity [115]. Magnusson et al. [148] designed the cultivation system to maximize the manipulation of the phenolic compounds without decreasing the seaweed growth rate. This study demonstrated the best technique for *Derbesia tenuissima* (Chlorophyta) (Figure 1N) cultivation to extract phenolic compounds of interest.

However, the potential observed by these studies demonstrates that there is still a long way to go until we can fully understand which drivers impact phenolic compounds’ quality and quantity and how to apply this knowledge in large-scale cultivation systems. Mostly, this phenolic compound bioactivity and yield are species dependent, thus for cultivation, there is the need to study the species beforehand in controlled cultivation systems.

Another way to explore this question is by mimicking the environmental conditions where the highest bioactivity was obtained and trying to obtain the data of all available parameters analyzed and studied. It can be faster to obtain the data, but it can be more difficult when trying the cultivation system. Nevertheless, if the seaweed is present in various geolocations with different results, this method can be a good alternative as a preliminary study before the seaweed cultivation assay, as described by Tanniou et al. [149].

## 4. Phenolic Compounds Application in Biotechnology

Biological compounds extracted from seaweeds exert several activities that can be exploited for the production of food, animal feed, and new drugs, substituting synthetic compounds with natural-origin compounds.

The most exploited phenolic compounds are phlorotannins, which are exclusively present in high concentration in brown seaweeds [17,124] and are involved in defense activities [150,151,152], showing strong antioxidant properties and antimicrobial activity, which help to inhibit bacterial growth [17]. Phlorotannins can be exploited in different biotechnological sectors. They exert a powerful antioxidant activity, as in the case of phlorotannins extracted from *Sirophysalis trinodis* (formerly known as *Cystoseira trinodis*, Phaeophyceae), which makes considering this species a potential source of phenolic compounds for diverse applications [153].

The synthesis of these compounds is driven by different factors. For example, seaweeds are particularly sensitive to external stressors; consequentially, they produce phenolic compounds, which develop multiple activities in order to protect seaweeds [153,154,155,156,157,158]. Due to several biological activities that involve phenolic compounds, they have been found interesting to be applied in the nutraceutical, pharmaceutical, medical, and industrial areas [34,75,159].

### 4.1. Medical and Pharmaceutical Applications

The consumption of seaweeds can prevent diseases or help the healing. Their bioactive compounds have positive effects on human health. For example, Tanniou et al. [149] identified the brown alga *Sargassum muticum* as a potential source of bioactive phenolic compounds: this species showed a strong antioxidant activity [150] and anti-proliferative activity in breast cancer cells [160] that may suggest the involvement of *S. muticum* in biotechnological applications.

Shibata et al. [161] compared the antioxidant activity of phlorotannins extracted from *Eisenia bicyclis* (Phaeophyceae) in vitro to available and active compounds such as vitamin C (ascorbic acid) and vitamin E (α-tocopherol). This study demonstrates that the antioxidant activity of phlorotannin was 10 times higher than that of other biological compounds.

The isolation and studies on phlorotannin derivates demonstrate that their high anti-proliferation activity is able to induce growth inhibition and apoptosis in human breast cancer cells [162,163]. For example, the red seaweed *Kappaphycus alvarezii* (also known as *Eucheuma cottonii*) (Figure 1J) polyphenol in vitro extracts were analyzed to evaluate antiproliferative, apoptotic, and cell cycle effects. Results showed an effect of these compounds against cancer cells [164].

The uptake of phlorotannins has also been related to the reduction in cardiovascular diseases and hypercholesterolemia [165,166].

Phlorotannins are responsible for the absorption of UV-B radiation [167,168,169,170], acting as photoprotective agent for algal cells [38,171], to avoid DNA damage [172,173,174]. This property is also effective for human and animal skin, reducing the probability of skin cancer due to UV-B radiation [168]. Additionally, phlorotannins prevent the production of matrix metalloproteinases (MMPs), enzymes that encourage the presence of wrinkles by degrading the extracellular matrix. For this purpose, seaweed phenolic compounds may be involved in the production of anti-aging creams and skin products [175].

Phlorotannins are also involved in the development of therapies to treat diverse allergic diseases. In Korean traditional medicine, phlorotannin extracts from the brown alga *Sargassum hemiphyllum* and the red alga *Polyopes affinis* (formerly known as *Carpopeltis affinis*) have been confirmed to have effective antiallergic properties in vitro [176]. The Japanese brown alga *Ecklonia arborea* (formerly known as *Eisenia arborea*) has been found to contain effective inhibitors of histamine; the presence of phlorofucofuroeckol B (phlorotannin) may be the reason for the anti-allergic activity shown in rats. *Ecklonia arborea* is popular in Japan since it has been consumed for years as healthy food and folkloristic therapies [177].

Among phenolic compounds, bromophenol and its derivates are widely investigated due to their potential activities. Studies conducted with *Leathesia marina* (formerly known as *Leathesia nana*) (Figure 1D) (Phaeophyceae) indicate that bromophenol derivatives respond positively to the inhibition of human cancer cells proliferation in vitro [178]. Alongside the ideal exploitation of bromophenol derivates for the development of new therapies for tumor treatment, these biological compounds reported antiviral activity against Herpes Simplex Viruses-1. For instance, extracts from the red alga *Symphyocladia latiuscula* (Figure 1N), which is abundant in Korea, demonstrate antiviral activity against HSV-1, likely due to the presence of its bromophenols, the major compounds [179].

Moreover, researchers have proven the antimicrobial effect of bromophenols extracted from the red alga *Rhodomela confervoides*, which act against some *Staphylococcus* and *Pseudomonas aeruginosa* strains [180].

#### Advantages of Phenolic Compounds Consumption for Human Health

Benefits of phenolic compounds are very common in human diet, since they can be ingested as food or food supplements and provide the human organisms with multiple positive effects [181]. They can be found in food and beverages from natural origin such as plants, seaweeds, fruits, coffee, black tea, and chocolate [182,183], but they can be also added to our daily diet as colorants or as antioxidants [184].

Many synthetic antioxidants have been developed to retard the oxidation in foods. However, synthetic compounds may have collateral effects [185] that could be avoided by the intake of natural antioxidant compounds, such as phenolic compounds extracted from seaweeds [186]. Phenolic acids present in food are also responsible for organoleptic properties, influencing color, flavor, and nutritional values [187].

Brown algae have already been exploited as food in Asia in the past 15 centuries; phlorotannin extracts from *Ecklonia cava* are already available in the market since 2018, when the European Food Safety Authority (EFSA) Panel on Dietetic Products, Nutrition and Allergies (NDA) attested that these extracts are indicated for diet due to their nutritional properties. *Ecklonia cava* thallus is consumed as salad and as a component of soups, while *E. cava* powder is also used to dye food, especially sweets, such as candies or rice cakes [188].

Phlorotannins have anti-diabetic effects: Roy et al. [189] assessed the in vitro inhibitory activity of phlorotannins extracted from *Ascophyllum nodosum* and *Fucus vesiculosus*, and their effect on rat blood glucose and insulin levels. It has been noticed that, 20 min after the consumption of animal feed enriched in phlorotannins, the normal increase in postprandial blood glucose was reduced by 90%, with a consequential reduction by 40% of insulin secretion [189].

As different classes of polyphenols from seaweeds can assure health benefits, it is suggested to consume the whole algae in order to uptake a higher quantity of bioactive compounds, instead of consuming only algae extracts as food supplements [190].

Flavonoids have been investigated for a long time for their powerful antioxidant activities. Their uptake has been linked with a reduced risk of lung cancer [181].

### 4.2. Aquaculture and Industrial Applications

Bromophenols are also investigated for the flavor they give to seafood [113,191,192]. Studies attested that bromophenols are responsible for the typical iodine-like flavor of marine fish [192], prawns, and marine algae [113]. It is quite likely that bromophenols detected in marine fish and prawns derived from their diet based on seaweeds that can synthesize these compounds [113,193].

The Japanese brown algae *Padina* spp., *Sargassum* spp., and *Lobophora* spp. (Figure 1E) have been detected as sources of bromophenols for local fish. It is likely that fish assimilate the typical marine flavor after the ingestion of these algae [69].

The presence of bromophenols in the diet of prawns may be useful for aquaculture [113,192]: crustaceans used as fish feed in aquaculture systems have low amounts of bromophenols due to their diet, with a consequential absence of iodine-like flavor in farmed fish [192]. The inclusion of seaweeds in prawns feed may thus increase the sea-like flavor of aquaculture seafood, enhancing their taste [69]

Moreover, other compounds, such as flavonoids, play an important role in retarding lipid oxidation that occurs in muscle, especially in fish, in order to delay the deterioration of seafood [186,194].

Over the last years, textile industries dedicated more attention towards medical textiles since their usage is not restricted to medical centers and care facilities: it is also present in other fields where hygienic conditions are required, e.g., hotels or restaurants [195]. Natural fibers such as cotton or silk are limited; therefore, medical textile industries started to use synthetic fibers, such as polyester, viscose, polyamides, and polypropylene [195]. A critical problem with synthetic fibers is the risk of spreading infections. To overcome this problem, seaweeds’ bio-compounds may be utilized for textile production. Due to the properties of phenolic compounds, new biological textiles may be developed. The new textiles could have antioxidant and antimicrobial properties [196] with the advantage of being natural and not irritating to the skin and being biodegradable and biocompatible [197,198]. The natural bioactive agents are non-toxic and skin and eco-friendly. From the extraction and treatment of cellulose-based polyphenols, these textiles can be brought into contact with the human skin and tissues and body fluids [195].

Moreover, the use of flavonoids to obtain UV-protective clothing has been suggested, since they show UV protection ability linked with antibacterial and anti-inflammatory properties [195].

## 5. Conclusions

This review highlights the diversity of phenolic compounds observed in seaweeds and their potential valorization in different application sectors.

Many polyphenols are exclusively synthesized by seaweeds, and, due to that, it is important to implement the knowledge about these compounds and their sources, in order to extract phenolic compounds that can be involved in the production of novel food, new drugs, and novel products.

Additionally, the use of natural compounds can diminish the use of synthetic ones which may compromise our health, while compounds extracted from natural sources may enhance our lifestyle and support disease prevention, but always through either a management of the natural resources or the establishment of algal culture to ensure a perennial sourcing of these phenolic compounds.

## Data Availability

Not applicable.
